# Toxicity-Induced Discontinuation of Immune Checkpoint Inhibitors in Metastatic Urothelial Cancer: 6-Year Experience from a Specialized Uro-Oncology Center

**DOI:** 10.3390/cancers16122246

**Published:** 2024-06-18

**Authors:** Severin Rodler, Can Aydogdu, Isabel Brinkmann, Elena Berg, Rega Kopliku, Melanie Götz, Troya Ivanova, Alexander Tamalunas, Gerald B. Schulz, Volker Heinemann, Christian G. Stief, Jozefina Casuscelli

**Affiliations:** 1 Department of Urology, University Hospital of Munich, 81377 Munich, Germany; 2 Comprehensive Cancer Center, University Hospital of Munich, 81377 Munich, Germany; 3 Department of Urology, University Hospital Schleswig-Holstein, 24105 Kiel, Germany; 4 Department of Internal Medicine III, University Hospital of Munich, 81377 Munich, Germany

**Keywords:** urothelial carcinoma, immunotherapy, long-term response, immune-related toxicity, discontinuation

## Abstract

**Simple Summary:**

This study analyzed patients with metastatic bladder cancer. A total of 108 patients received immunotherapy, but 11 had to stop their immunotherapy because of adverse events to the therapy. Despite stopping the therapy, the treatment effect could be seen for a longer time, and many patients did not have to start therapy again. This study helps us to understand what happens when patients have to stop immunotherapy due to adverse events and what to do next regarding cancer therapy for those patients.

**Abstract:**

Immune checkpoint inhibitor (ICI) therapies have been established as the standard-of-care in various uro-oncological cancers. Immune-related adverse events (irAEs) are frequent, but their degree rarely leads to the discontinuation of immunotherapies. Unplanned permanent treatment discontinuation may negatively impact the outcomes of patients, but there are emerging data about a positive correlation between emergence of severe irAEs and therapeutic cancer responses. In this study, a retrospective analysis of patients treated for urothelial carcinoma (UC) with ICI-based immunotherapy was conducted. irAEs were classified according to the Common Terminology Criteria for Adverse Events (CTCAEs) and radiological responses according to the Response Evaluation Criteria In Solid Tumors (RECISTs). Out of 108 patients with metastatic urothelial cancer that underwent immunotherapy, 11 experienced a severe irAE that required permanent discontinuation of ICI therapy. The most frequent irAEs leading to discontinuation were hepatitis (*n* = 4), pneumonitis (*n* = 2), and gastritis or colitis (*n* = 2). Prior to discontinuation (R1), the radiological best response was complete remission (CR) in three patients, partial response (PR) in six, and stable disease (SD) in wo patients. After the discontinuation of ICI therapy (R2), the best responses were CR in six, PR in three, and SD in two patients. Following discontinuation, the majority of these patients showed a sustained treatment response, despite not receiving any cancer-specific treatment. The median time of response after discontinuation of ICI therapy was 26.0 (5.2–55.8) months. We propose accurate counseling and close follow-ups of patients following their discontinuation of ICI therapy due to irAEs, as responses can be durable and deep, and many patients do not require immediate subsequent therapies, even in urothelial cancer. More data are required to find predictors of the length of response to appropriately counsel patients.

## 1. Introduction

The advent of immune-based therapy regimes has changed the treatment landscape of various types of cancers due to their potential durable responses and mostly low-grade toxicities compared to cytotoxic chemotherapy [1]. Immune checkpoint inhibitors (ICIs) like programmed cell death protein 1 (PD-1) antibodies such as pembrolizumab [2] or programmed death-ligand 1 (PD-L1) antibodies such as atezolizumab [3] have thereby revolutionized the therapy of urothelial carcinoma (UC), including adjuvant treatment [4] and maintenance therapy with the PD-L1 antibody avelumab following cytotoxic chemotherapy [5]. However, metastatic urothelial cancer is still a very deadly cancer, and the number of responders to ICIs is limited as complete remissions are exceptions [3]. Currently, the field is expanding towards earlier application of ICIs during the disease course and combination regimens with other drug classes as antibody–drug conjugates [6]. Putatively, the trend will provide additional efficacy, but safety profiles may change [7].

Immune-related adverse events (irAEs) are autoimmune conditions triggered by ICIs and can affect any organ in the body. They exhibit different biological characteristics, any onset time and duration, and various levels of severity [8]. IrAEs can occur at any time during a patient’s treatment course, most commonly in the first couple of months of treatment, but in some cases, even well after treatment discontinuation. In uro-oncology, irAE rates are 81.6% for any grade across 92 clinical studies conducted since their initial launch [9]. While generally low-grade, most irAEs allow continuation of the ICI therapy, combined with tailored symptom management. More severe irAEs are rare and require withholding the ICIs and treatment with systemic glucocorticoids or other immunosuppressants. Whether the restart of ICI is recommenced depends on the degree of the irAE and the damage to the affected organ [10]. Several previous studies on non-small-cell lung cancer, gastric cancer, and UC have associated the incidence of irAE with a good therapeutic response to ICIs [11].

However, permanent discontinuation of immunotherapy due to higher-grade toxicity is rare, and the management of those patients is unclear, and their clinical outcomes are not clearly described. We therefore hypothesize that patients with irAEs that lead to discontinuation experience durable responses in a real-world setting.

## 2. Materials and Methods

Patients with metastatic urothelial carcinoma (mUC) were treated at the Munich *Comprehensive Cancer Center* (CCC), one of fourteen highly specialized interdisciplinary oncology centers in Germany. Here, patients have access to high-quality oncological treatment as well as supportive care such as psycho-oncological and nutrition counseling, physical activity, and pain treatment. Dedicated patient education about onset, detection, and therapeutic options in cases of irAEs was performed. Prior to the initiation of this study, the institutional review board approved the study design (reference number: 19-942). All patients provided written informed consent prior to their enrollment in this study. The findings of this study have been reported according to the *Strengthening the Reporting of Observational Studies in Epidemiology* (STROBE) checklist (Supplementary Table S1) [12].

Patients with genitourinary cancer are prospectively enrolled into a database and followed-up during treatment visits or via telemedical consultations, as previously described [13]. Patients received pembrolizumab for first-line therapy in cisplatin-ineligible patients, depending on PD-L1 expression measured by the *Combined Positive Score* (CPS ≥ 10). Alternatively, patients received pembrolizumab as their second-line therapy after first-line chemotherapy, according to the guidelines of the European Association of Urology (EAU) [14].

All irAEs were classified by specialized uro-oncologists prior to treatment administration. Grading was performed according to the *Common Terminology Criteria for Adverse Events* (CTCAEs). Further treatments were performed according to the *European Society for Medical Oncology* (ESMO) guidelines [10] and its later amendments as well as the current EAU guidelines for mUC [7].

**Figure 1:**
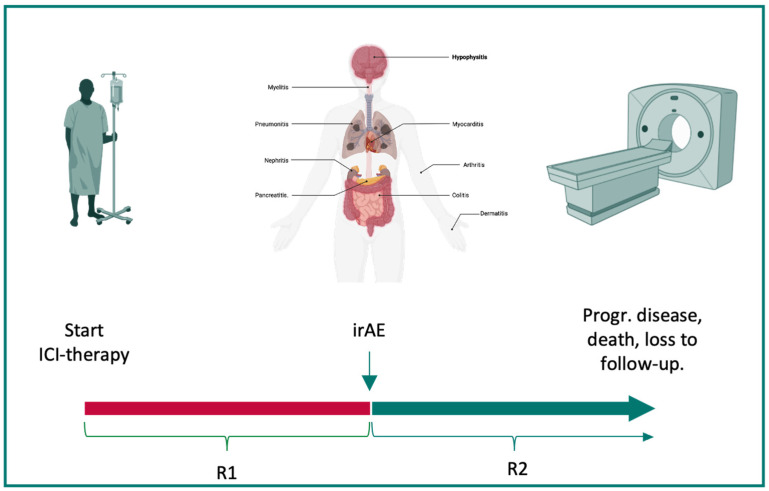
Balancing irAEs and responses to ICI therapy. Severe immune-related adverse events (irAE) can lead to the discontinuation of immunotherapy. Therefore, the time between the initiation of immune checkpoint inhibitor (ICI) therapy and discontinuation due to irAEs was defined as R1 (red). The time between the occurrence of an irAE and progressive disease, death, or loss to follow-up was defined as R2 (green). The best response during both time frames was assessed via CT imaging. This figure was created with www.BioRender.com. Abbreviations: ICI: immune checkpoint inhibitor therapy, irAE: immune-related adverse event, progr: progressive, R1: response 1, and R2: response 2.

Radiological assessments were performed by board-certified radiologists. As the standard imaging modality, a CT of the thorax and abdomen with a contrast agent was performed. Radiological responses were classified according to the recent version of the *Response evaluation criteria in solid tumors* (RECISTs) guidelines [15]. Response 1 (R1) was defined as the best response observed between the start of ICI therapy and discontinuation, and response 2 (R2) was defined as the best response between discontinuation and progressive disease, loss to follow-up, or death. Duration of response was assessed as the time frame between the respective time points. Loss to follow-up was defined as a patient not providing data for the study endpoint of R2 without reaching the death endpoint (see Figure 1).

Patients were closely followed-up after treatment discontinuation. They continued to have access to the comprehensive cancer center and its associated facilities. Follow-up visits included radiological response assessments and assessments of irAEs. Treatment decisions regarding further discontinuation of ICI therapy were performed on a patient-by-patient basis, carefully considering comorbidities, current radiological responses, and resolution of irAEs. These decisions, along with therapeutic alternatives and potential outcomes, were communicated in detail with the patients to ensure shared decision-making, enhance adherence to follow-up, and reduce anxiety. Patient education about irAEs was continued, and patients were made aware of the potential for the late onset of further irAEs even after their discontinuation of ICI therapy.

## 3. Results

A total of 11 out of 108 patients with urothelial cancer and at least one dose of ICI therapy treated at our academic center between 2017 and 2023 developed irAEs requiring permanent discontinuation of ICI therapy. The median age was 73 (43–87) years, and the median follow-up time was 34 (17–63) months. All eleven patients received pembrolizumab as either their first- (*n* = 5) or second-line therapy following chemotherapy (*n* = 6). 

The most frequent irAEs leading to discontinuation of ICI therapy were hepatitis (*n* = 4), pneumonitis (*n* = 2), and gastritis or colitis (*n* = 2). Four patients developed more than one irAE at their time of discontinuation. IrAE treatment required oral corticosteroids (*n* = 11) or escalation to mycophenolate mofetil (*n* = 1). Five patients required corticosteroids for more than 3 months. irAEs completely resolved after a median of 4 (3–9) months. Patient baseline characteristics are listed in Table 1. 

Interestingly, four patients developed more than one irAE at the time of discontinuation of ICI therapy. Three patients experienced hepatitis with either giant cell arteritis, thyreoditis, or myositis. One patient presented with gastritis and dermatitis. The patient experiencing giant cell arteritis and hepatitis at the time of discontinuation required mycophenolate mofetil in addition to prednisolone. 

**Figure 2:**
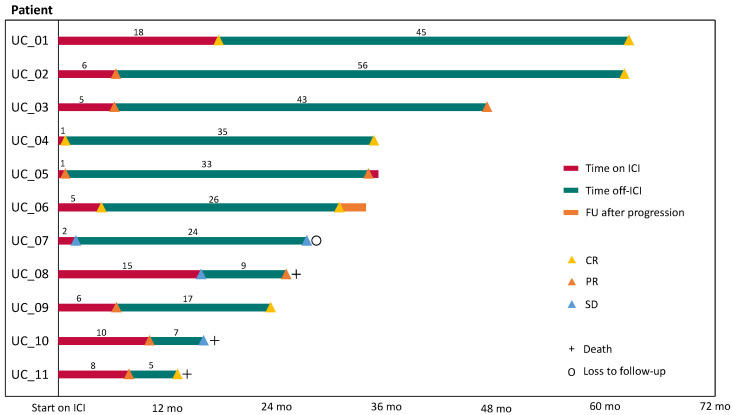
Duration of response in patients with discontinuation of ICI therapy due to severe irAEs. The patient’s response prior to and after their cessation of immunotherapy was assessed. Response 1 (R1, red) was defined as the time between the start of immunotherapy and discontinuation. Response 2 (R2, green line) was defined as the time from discontinuation to progressive disease, death, loss of follow-up, or last visit. The months of R1 and R2 are rounded to full months. Abbreviations: UC, urothelial carcinoma; ICI, immune checkpoint inhibitor; FU, follow-up; CR, complete remission; PR, partial remission; and SD, stable disease.

The best response prior to irAEs (R1) was complete remission (CR) in three patients, partial response (PR) in six patients, and stable disease (SD) in two patients. After discontinuation of ICI therapy (R2), the best observed radiological response was CR in six, PR in three, and SD in two patients. Thereby, three PRs progressed to CR after discontinuation. The median duration of R1 was 6.4 (0.7–17.7) months, and the median duration of R2 was 26.0 (5.2–55.8) months. Three patients progressed during their time off ICI therapy. Patient UC_05 progressed after 33 months off ICI therapy, and patient UC_06 after 26 months. UC_05 has been re-exposed to ICI therapy (pembrolizumab) under close observation without experiencing any irAEs until their last follow-up. Patient UC_06 experienced metachronous UC of the bladder after metastatic upper tract urothelial carcinoma. This patient was subsequently treated with palliative radiotherapy for local tumor control in the bladder. UC_10 revealed rapid progression 6 months after discontinuation of ICI therapy and died due to their poor condition and advanced age at the time of progression (Figure 2).

## 4. Discussion

There are emerging data on patient-favorable outcomes following severe toxicity-induced discontinuation of ICI therapy, but the management of those patients remains an understudied phenomenon [16]. Uncertainty predominates regarding the surveillance and re-challenge of ICI upon resolution of the irAE, in particular if a complete response was not achieved or after a very early onset of toxicities [17]. Tumor-specific experience and guidance would be helpful for management, but the extent, outcomes, and implications of irAEs are highly unpredictable due to the initiated immune process. We provide insights into the real-world course of patients experiencing irAEs that lead to discontinuation of therapy and reveal durable responses in the majority of patients. 

Discontinuation of ICI therapies has been controversially discussed since the first approval of this class of drugs. In non-small-cell lung cancer, discontinuation following either a response or stable disease seems to provide sustainable benefits [18]. Another concept has arisen: interest in the field of ICI therapies for melanoma. Here, cessation following response is seen as safe and shows sustained benefit when patients had ongoing responses after 1 or 2 years [19]. A similar picture is seen in patients with metastatic RCC and responses to therapy. Here, the cessation of ICIs after a predefined cumulative ICI dose revealed cases with durable responses in different clinical trials [20]. But the duration of ICI treatment has not been definitely elucidated, and particularly for urothelial cancer, there are no data concerning this matter. Based on our data, there is evidence to further focus on this matter in future studies.

In our institution, patients only discontinue ICI treatment due to progression of disease or, as described here, due to severe irAEs. Therefore, the central issue is whether or when to continue with further treatment lines due to the latter reason. 

After discontinuation, patients in our cohort continued to undergo radiological response assessments every 3 to 4 months. Patients were not started on a further therapy line unless they revealed progressive disease. However, they were provided with continuous access to the resources of a comprehensive cancer center and were regularly counseled [21]. From our experience, patients accept not to be on treatment when being closely monitored and counseled regarding the effects of ICI therapies and irAEs. This counseling is important for two aspects: adherence to follow-up to detect potential recurrence or progression as early as possible and to detect the late onset of irAEs. Late onset of irAEs is understudied in clinical trials [22], but they can occur even after the cessation of immunotherapy [23]. Therefore, real-world data from our study and other studies might improve patient counseling in those difficult situations and provide the evidence for current recommendations.

Tailoring uro-oncological therapies to balance the economic burden for patients and healthcare systems, side effects, and the efficacy of anticancer treatment is important and a subject of a continuous debate in urology [24]. UC is a malignant entity where patients are affected by financial toxicity in particular [25]. Therefore, treatment deintensification in highly selected patients might be an option. We observed that most patients that initially responded to the ICI treatment revealed deep and durable responses after irAE-related discontinuation. However, two patients revealed still progressive disease after about 2 years off ICI therapy. Longer follow-ups and larger cohorts are required to identify predictors for durable responses in patients after discontinuation of immunotherapy. 

Biomarkers to predict responses and irAEs have been tested in clinical trials but have not reached clinical practice due to conflicting or unclear results [26]. Interestingly, the patient’s microbiome seems to play a role in efficacy and irAE development under ICI therapy [27,28]. However, it remains uncertain whether these mechanisms represent a correlation, where the microbiome acts merely as a biomarker for the overall activity of the immune system, or if the microbiome is mechanistically involved in modulating these effects [29]. Therefore, the exploration of biomarkers and a deeper understanding of the underlying physiology are of high importance for future developments in ICI therapy administration. Interestingly, we observed hepatitis in three cases, where patients experienced more than one irAE that led to discontinuation. Future large-scale studies also have to look into the specific patterns of irAEs that lead to discontinuation, potentially with hepatitis in addition to other irAEs being the most frequent ones.

Several groups have hypothesized that the immune activation mechanisms that determine most irAEs might be coupled with the activity required for antitumor immune responses [30]. These mechanisms are currently not entirely understood on the molecular level. However, the entire PD-1/PD-L1 pathway is a highly important regulator to control overactivity and limit the response of the immune system [31]. We were able to reproduce the hypothesis in our observation of urothelial cancer treatment. This tumor, with a mostly modest response to ICIs compared to other entities, has shown durable responses to treatment following severe irAEs.

The management of irAEs goes beyond discontinuation of ICI therapy, steroid dosage adjustments, and potential re-exposure. Patient counseling and close follow-ups are of high importance and can potentially improve adherence in challenging treatment situations, as observed in our study. It is crucial not only to react to irAEs once they appear but also to prevent them. However, constant monitoring is time-intensive and may not be easily performed outside academic centers. Therefore, new technology is needed to detect irAEs early and actively educate patients. Digital therapeutics and wearable technologies have been identified as potential tools to fill this gap in uro-oncology. [32,33]. These tools are primarily focused on tailored tracking of irAEs via digital biomarkers, assessing them in digital diaries, providing expert recommendations for irAE therapy, and offering techniques to manage anxiety arising from therapy discontinuation. Future applications will help advance immune checkpoint inhibitor (ICI) therapies to more remote settings.

New therapeutic combinations are being added to the treatment armamentarium for patients with mUC. Antibody–drug conjugates (ADCs) targeting Nectin cell adhesion molecule 4 (Nectin-4) or Trophoblast cell surface antigen-2 (Trop-2) have been identified as novel therapeutic options [34,35]. Combination therapies, in particular, have significantly altered the therapeutic landscape. Pembrolizumab in combination with enfortumab vedotin, a Nectin-4-targeting ADC, has demonstrated remarkable efficacy, has been approved as a first-line therapy for patients with mUC by the *U.S. Food and Drug Administration* (FDA), and is under consideration by the *European Medical Agency* (EMA) [36,37]. However, combination therapies present overlapping toxicity profiles that must be managed by uro-oncologists, posing significant treatment dilemmas [36]. In our study, five patients received pembrolizumab as their first-line therapy. With the current shifts in front-line treatment, monotherapy without enfortumab vedotin can be expected to become rare. Nevertheless, data on monotherapies are urgently needed to understand the long-term benefits for patients with severe irAEs and determine whether to continue ICI therapy. Therefore, this study provides unique data crucial for evaluating the isolated effects of single therapies and applying those findings to the treatment of patients with combination therapies.

Counseling patients in this challenging treatment situation of discontinuation is difficult and is based on an established patient–physician relationships. With the identification of more biomarkers, but also with the data from the current study, counseling might be improved in the near future, and treatment algorithms can be adopted based on our findings. As patients trust in the active use of technology in the process of counseling in complex decisions in uro-oncology [38], the addition of more complex diagnostic tools than currently applied will mark a major step forward. New biomarkers based on different artificial intelligence applications are currently being investigated to predict responses to immunotherapy [39] and adverse events [40]. Advances in technology, such as the introduction of generative AI, might further improve not only the prediction and detection of irAE but also the communication with patients regarding their treatment [41]. These findings might reshape the treatment landscape and decision-making processes in uro-oncology.

This retrospective analysis is limited by the relatively high baseline response rates in selected patients that may be associated with the durable responses observed, despite treatment cessation. Second, the overall limited number of patients and single-center design might bias the results and overestimate the effects and frequency of events observed in this study. Hence, further real-world data are required as obtained for the enfortumab vedotin monotherapy in multicentric settings [42]. Selection bias might apply as patients were treated by physicians’ choice, and therefore favorable patients might be selected for this treatment approach. In addition, extended durations of the therapeutic effects of ICIs surpass their pharmacokinetic half-lives and promote persistent pharmacodynamic effects (clinically manifested as durable responses). But putatively, these patients develop immunological memory, which might also have implications for broader and longer-term T cell-mediated toxicity. The positive outcome may be linked to the incidence of severe toxicities. Further studies are urgently needed to unravel the underlying mechanisms of the correlation and promote a tailored approach to patients. 

## 5. Conclusions

Discontinuation of ICI therapy due to irAEs is a phenomenon that is observed in patients with genitourinary cancer. Despite discontinuation, patients with initial responses to ICI therapy experienced durable and deep responses, even in urothelial cancer, as observed in this study. As high-grade immunotoxicity is a potential biomarker for responses observed in other studies, more data are urgently required to counsel patients experiencing this challenging treatment situation.

## Figures and Tables

**Table 1:** Clinical characteristics of patients with irAEs that led to discontinuation.

Variables	Urothelial Carcinoma (*n* = 11)	
Age		
Median	73	
Range	43–87	
Follow-up		
Median	34	
Range	17–63	
	n	%
Sex		
Male	6	54.5
Female	5	45.5
Therapy line		
1st line	5	45.5
2nd line	6	54.5
Grade 3–4 irAEs ^1^		
Hepatitis	4	36.4
Pneumonitis	2	18.2
Gastritis/colitis	2	18.2
Arthritis	1	9.1
Myositis	1	9.1
Myocarditis	1	9.1
Thyreoiditis	1	9.1
Fatigue	1	9.1
Dermatitis	1	9.1
Giant cell arteritis	1	9.1
Management of irAEs		
Corticosteroids	11	100.0
MMF	1	9.1
Duration of irAE, months		
Median	4	
Range	3–9	

Abbreviations: ICI: immune checkpoint inhibitor, irAE: immune-related adverse event, and MMF: mycophenolate mofetil. ^1^ Patients experience more than one irAE at the time of discontinuation.

## Data Availability

The dataset will be made available on request from the authors.

## Supplementary Materials

The following supporting information can be downloaded at: https://www.mdpi.com/article/10.3390/cancers16122246/s1, Table S1. STROBE Statement—Checklist of items that should be included in reports of cohort studies.
